# Enhanced Surface Properties of Carbon Fiber Reinforced Plastic by Epoxy Modified Primer with Plasma for Automotive Applications

**DOI:** 10.3390/polym12030556

**Published:** 2020-03-03

**Authors:** Kyeng-Bo Sim, Dooyoung Baek, Jae-Ho Shin, Gyu-Seong Shim, Seong-Wook Jang, Hyun-Joong Kim, Jong-Won Hwang, Jeong U. Roh

**Affiliations:** 1 Laboratory of Adhesion and Bio-Composites, Program in Environmental Materials Science, Seoul National University, Seoul 08826, Korea; skb181@snu.ac.kr (K.-B.S.); baek.s.dy@snu.ac.kr (D.B.); pass2462@snu.ac.kr (J.-H.S.); sks6567@snu.ac.kr (G.-S.S.); jangsw0202@snu.ac.kr (S.-W.J.); 2 Research Institute of Agriculture and Life Sciences, College of Agriculture and Life Science, Seoul National University, Seoul 08826, Korea; 3 Kukdo Chemical Co., Ltd, Gasandigital 2-ro, Seoul 08588, Korea; pacs@kukdo.com; 4 Gumi Electronics & Information Technology Research Institute, Gumi 39171, Korea; njwice@naver.com

**Keywords:** epoxy, CFRP, lightweight, automobile, surface treatment

## Abstract

Carbon fiber reinforced plastic (CFRP) is currently used as a lightweight material in various parts of automobiles. However, fiber reinforced plastic (FRP) material may be damaged at the time of joining via mechanical bonding; therefore, adhesion is important. When bonding is conducted without surface CFRP treatment, interfacial destruction occurs during which the adhesive falls off along with the CFRP. Mechanical strength and fracture shape were investigated depending on the surface treatment (pristine, plasma treatment times, and plasma treatment times plus epoxy modified primer coating). The plasma treatment effect was verified using the contact angle and X-ray photoelectron spectroscopy. The wettability of the epoxy modified primer (EMP) coating was confirmed through surface morphology analysis, followed by observation of mechanical properties and fracture shape. Based on test data collected from 10 instances of plasma treatment, the EMP coating showed 115% higher strength than that of pristine CFRP. The adhesive failure shape also changed from interfacial failure to mixed-mode failure. Thus, applying an EMP coating during the automotive parts stage enhances the effect of CFRP surface treatment.

## 1. Introduction

The automotive industry is currently focusing on the use of lightweight materials (such as composite materials, aluminum, magnesium, and plastics) to increase energy efficiency and reduce CO_2_ emissions. In general, a weight reduction of 100 kg corresponds to a CO_2_ emission reduction of 7.5–12.5 g/km. Therefore, weight reduction is becoming increasingly important in the automotive industry. Carbon fiber reinforced plastics (CFRPs) are used in various fields, including the aviation and automotive industries. The greatest advantages of CFRP materials are their light weight, high strength, corrosion resistance, and high rigidity [1,2]. 

Due to high cost, production processing issues, and restricted functionality, CFRP should be used with steel or other similar materials. Existing physical joining methods, such as riveting, bolting, and punching, are unsuitable for fiber-reinforced plastics (FRPs). Because FRP comprises multiple layers, it is prone to cracking around such physical joints, which causes significant damage to the substrate owing to propagation of these cracks [3,4]. Stress concentration is another major issue during physical joining as stress concentrates around bolts and rivets, leading to failure in these areas. Stress concentration is more severe for FRPs than for metals [5,6,7]. 

A solution for stress concentration is adhesive bonding. When using adhesives, stress is distributed evenly between the substrate and the surface of the substrate, thus avoiding the problem of stress concentration [8,9]. 

CFRPs are manufactured by various methods such as prepreg compression molding (PCM), CFRP, and autoclave. In this case, silicone release agents are used to facilitate demolding. However, these release agents remain on the PCM CFRP surfaces, interfering with chemical bonding and adhesion [10,11,12]. This induces surface irregularities via resin delamination during demolding.

Proper pretreatment of a surface is necessary to improve adhesion performance. Pretreatment includes methods of removing surface contaminants, removing oxidized layers, improving wettability, chemical modification, and changing the surface roughness. Typically used surface treatment methods include abrasion and blasting for directly treating the surface. This allows for a low cost and simple surface treatment. The disadvantage is that it not only damages the surface resin but also the fiber, which can cause additional reliability problems and requires additional cleaning [13]. Another surface treatment method is the use of an excimer laser. This method selectively removes surface contaminants with little damage to the fibers. Depending on the wavelength and radiation, the surface cutting can be adjusted [11]. Although laser processing can be delicate, there are many limitations to its applications in automotive processes due to equipment cost. Among these surface treatments, plasma treatment induces chemical changes such as forming oxygen-containing groups on the surface; the surface roughness also shows changes such as smoothing [14,15]. The oxygen-containing groups on the surface can improve the surface wettability and adhesion performance, also increasing surface energy [16]. The plasma surface treatment process is also simple and relatively easy to introduce.

In this study, an experiment was conducted in which a coating was formed on the activated CFRP surface after plasma treatment to create a single coating layer with strong chemical bonding. We investigated the chemical bonding strength depending on the number of plasma treatments. The effect was verified by evaluating the surface morphology and mechanical properties. The adhesion strength between the PCM CFRP and the coating layer was confirmed using a surface and interfacial cutting analysis system (SAICAS). After measuring the mechanical strength, the effect of the coating was confirmed by observing the fracture shape of the CFRP.

## 2. Materials and Methods 

### 2.1. Materials

The specimens used were PCM CFRP and steel plate. The PCM CFRP was composed of an epoxy resin matrix and plain weave prepreg (WSN-3KT, SK Chemicals, Seoul, Republic of Korea). The SK Chemicals prepreg was of a snap curing type. The PCM CFRP laminated 13 prepreg layers and laid up to 0° and 90°. The laminates were cured in a compression molder at 150 °C for 5 min under a pressure of 2.0 MPa. The steel plate type used was cold rolled steel (CR340, Posco, Pohang, Republic of Korea); CR340 is widely used in pillar parts in the automotive industry.

#### 2.1.1. Epoxy Modified Primer 

The epoxy modified primer (EMP) used YD-128, Dyhard 100 s, and Dyhard UR500. YD-128 (Kukdo Chemical, Seoul, Republic of Korea) is a bisphenol-A epoxy resin. The curing agent (Dyhard 100 s, Alzchem, Trostberg, Germany) and latent curing agent (Dyhard UR500, Alzchem, Trostberg, Germany) were blended with YD-128 and ethyl acetate according to an equivalent ratio of 1:0.7:0.6 of base epoxy, curing agent, and latent curing agent, respectively. Ethyl acetate was used to adjust the viscosity at the blending weight ratio of 18%. Detailed information on the materials used in this work is listed in Table 1.

#### 2.1.2. Plasma Treatment

An atmospheric pressure plasma unit (PLAMI-α, APP, Hwaseong, Republic of Korea) was used for CFRP surface activation with Ar and oxygen as the active gases at a flow rate of 10 mL/min. Plasma treatment was performed at a height of 5 mm and output power of 100 W. The exposure time for one cycle of plasma was 10 s. The surfaces were treated with 0, 1, 5, and 10 plasma treatment cycles. This permitted increases in the ratio of oxygen-containing groups.

### 2.2. Epoxy-Modified Primer Coating

**Figure 1:**
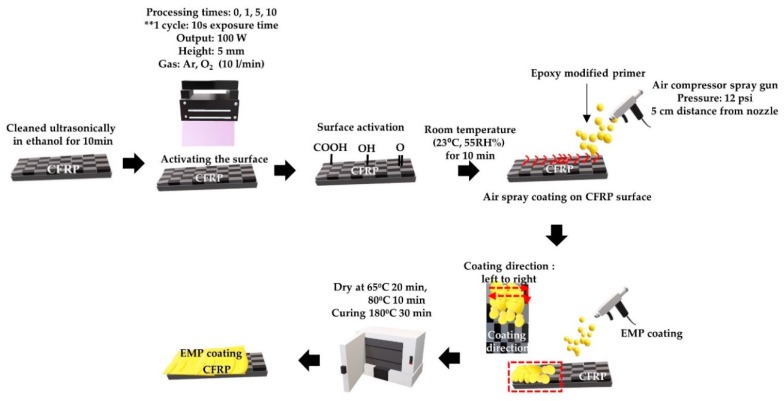
Schematic of epoxy modified primer (EMP) coating process.

Figure 1 shows Epoxy-Modified Primer Coating process. EMP coating was carried out in the following manner: The CFRP surface was ultrasonically cleaned in ethanol for 10 min to remove the release agent used during thermos-compressing. Plasma treatment was conducted for surface activation. After 0, 1, 5, or 10 plasma treatment cycles on the CFRP surface, the CFRP was maintained at room temperature (23 °C, 55% RH) for 10 min. EMP coating was performed on the activated CFRP surface. Using an air compressor spray gun at a pressure of 12 psi (82 kPa), 5 cm from the gun nozzle, the coating was applied from left to right. After EMP coating, the drying and curing steps were conducted in an oven at 65 °C for 20 min and 80 °C for 10 min. After the ethyl acetate dried, curing was performed at 180 °C for 30 min. It was then cooled to room temperature (23 °C, 55% RH). Figure 1 shows the process of EMP coating.

### 2.3. Contact Angle

The wettability according to the number of plasma treatments was confirmed through contact angle measurements (Phoenix 150, SEO, Suwon, Republic of Korea) using 2 µL deionized water droplets. The temperature was maintained at 25 °C while the relative humidity was 55%.

### 2.4. X-Ray Photoelectron Spectroscopy

X-ray photoelectron spectroscopy (XPS; AXIS SUPRA, Kratos, Manchester, UK) was used to verify the state of change of activated groups after plasma treatment. The XPS system used an Al Kα radiation source for surface chemical analysis.

### 2.5. Surface Analysis

A field-emission scanning electron microscope (FESEM, SIGMA, Gillingham, U.K.) and noncontact 3D surface profiler (Nano View-E1000, Nano System, Daejeon, Republic of Korea) were used to examine the surface morphology changes of the EMP coating after plasma treatment. 

### 2.6. Surface and Interfacial Cutting Analysis System

**Figure 2:**
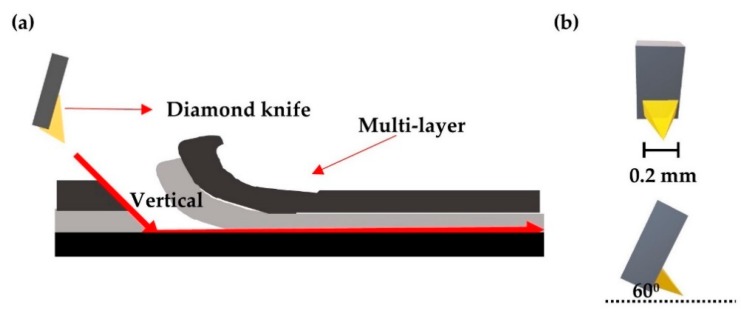
Schematic of (**a**) surface and interfacial cutting analysis system (SAICAS) operation, (**b**) cutting blade.

A SAICAS (SAICAS EN-EX, Daipla Wintes, Saitama, Japan) was used to directly evaluate the degree of bonding between the CFRP surface and the coating layer using a 0.2 mm wide diamond blade. Figure 2 shows that the coating was first cut in the vertical direction and then in the horizontal direction. Therefore, the peeling force exerted by the coating on the CFRP surface was confirmed.

### 2.7. Lap Shear Test 

**Figure 3:**
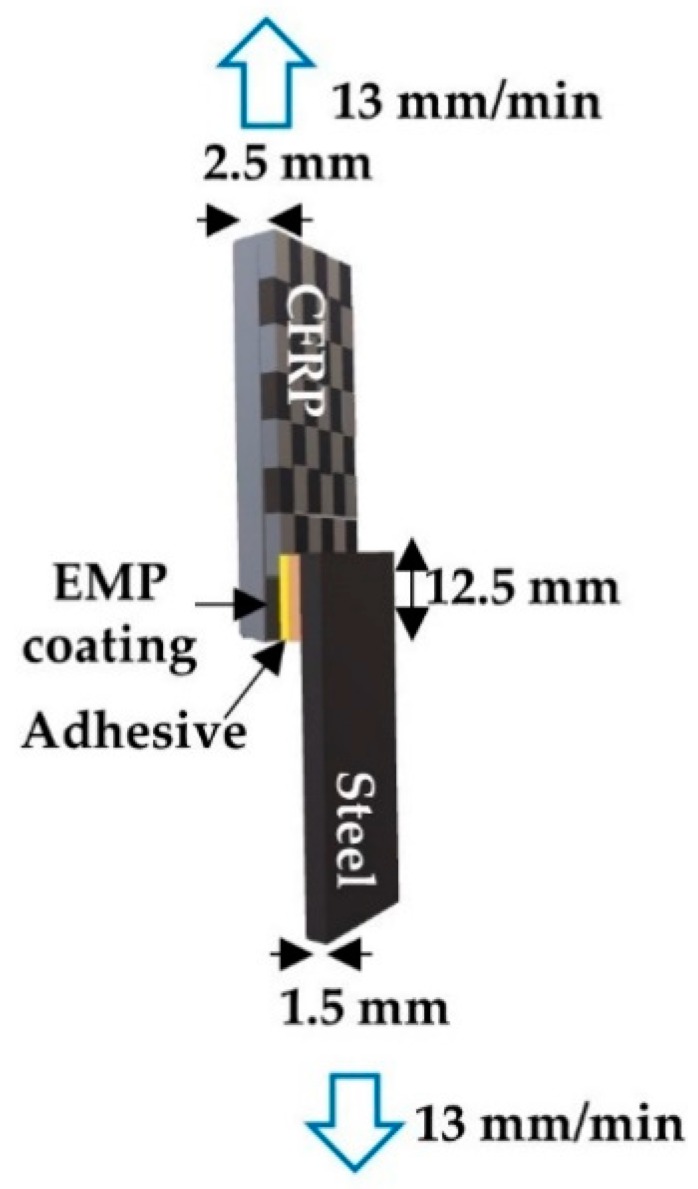
Schematic of testing lap shear strength of dissimilar materials.

The lap shear test was performed using a universal testing machine (Allround Line Z010, Zwick, Ulm, Germany) to confirm the EMP coating effect. Figure 3 shows that the CFRP specimen was 25 × 100 mm^2^ and was 2.5 mm thick, whereas the CR340 steel plate specimen was of the same width and length but was 1.5 mm thick. The adhesives used were KSR-177 (Kukdo Chem, Seoul, Republic of Korea) and G5022 (Kukdo Chem, Seoul, Republic of Korea) with an equivalence ratio of 1:1.1. Detailed information regarding the materials used in this work is listed in Table 2. The thickness of the adhesive was controlled using a 200 µm shim stock and applied over an area of 25 × 12.5 mm^2^.

## 3. Results and Discussion

When manufacturing PCM CFRP, a silicone release agent was applied to the mold for easy demolding. This release agent remained as a contaminant on the PCM CFRP surface. Such contaminants reduce adhesive performance [17]. Therefore, surface treatment is often required.

### 3.1. Surface Modification through Plasma Treatment

**Figure 4:**
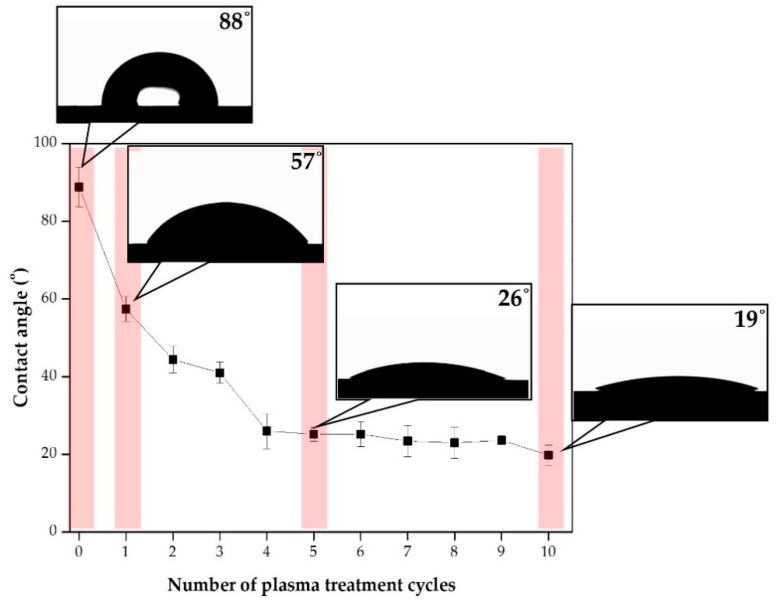
Contact angle according to the number of plasma treatment cycles.

Plasma treatment was conducted to activate the surface. The generation of oxygen functionalities can improve the wettability of PCM CFRP. Figure 4 shows that the nature of the CFRP changed from hydrophobic to hydrophilic depending on the number of plasma treatment cycles. The contact angle with no plasma treatment was 86°. Wettability increased sharply with increasing numbers of applied plasma treatments (0, 1, 5, 10 times); however, Figure 4 shows that the wettability remained very low after four cycles were applied. The contact angle gradually decreased and stabilized after four treatment cycles. The experimental numbers of plasma treatments were 0, 1, 5, and 10. 

**Figure 5:**
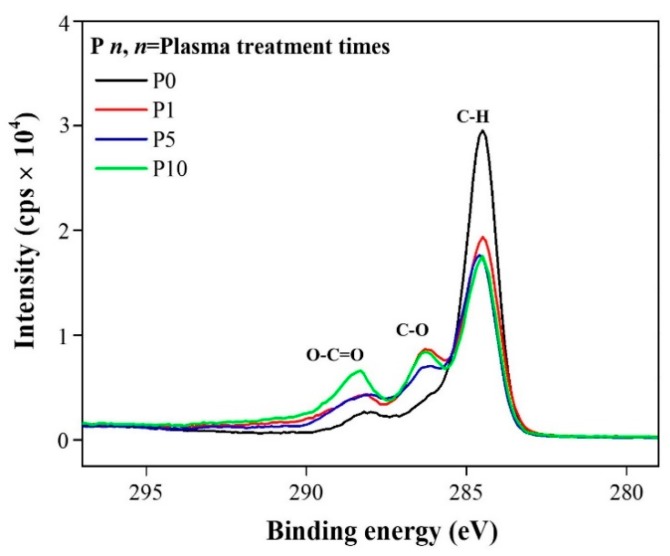
C1s X-ray photoelectron spectroscopy data of coatings with different numbers of plasma treatment cycles.

The activated surfaces after 0, 1, 5, or 10 plasma treatment cycles were examined by XPS. The surface composition was observed to shift following plasma treatment. High-resolution spectra of C1s showed that the peak intensities of the oxygen functional groups were dramatically changed for three components (284.6, 286.2, and 289.2 eV). The peak at 284.6 eV represented hydrocarbons (C–H), the peak at 286.2 eV represented alkoxy groups (C–O), and the peak at 289.2 eV represented carboxyl groups (O–C=O) [18]. The amount of oxygen increased with increasing numbers of plasma treatments; as a result, the peak intensities of the oxygen functional groups also increased [19]. Figure 5 shows that as the number of plasma treatments increased, the hydrocarbon peak intensity decreased while those of the alkoxy group and carboxyl group increased. The increased carboxyl group intensity indicated crosslinking with the epoxy [20].

### 3.2. EMP Coatings

Plasma-treated materials are difficult to store for long periods because they show inferior endurance and, over time, they return to their original energy states [21]. To solve this problem of unsustainability of plasma treatment, we used EMP coatings. 

**Figure 6:**
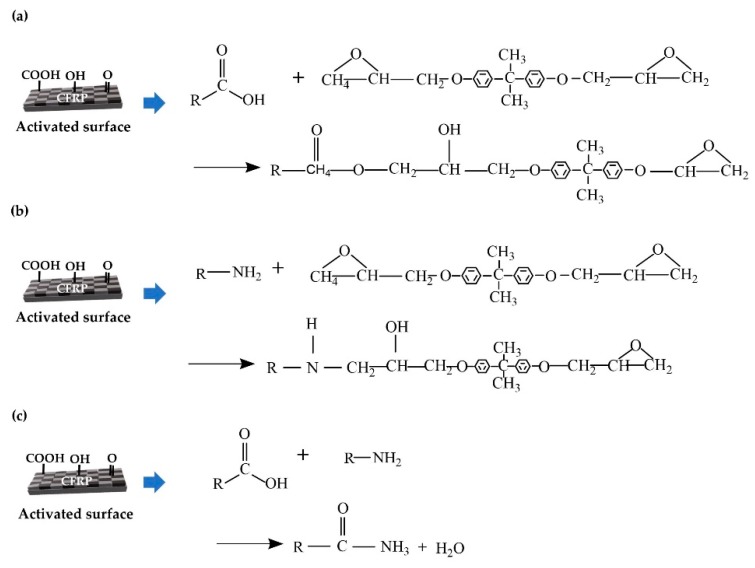
Chemical reaction between (**a**) carboxyl group and epoxy, (**b**) amine and epoxy, and (**c**) carboxyl group and amine.

EMP coatings were prepared by blending an epoxy, curing agent, latent curing agent, and solvent. However, the EMP coating is not simply a single-layer coating. The most important element in EMP coating is the chemical bonding of epoxides and amines with activated groups generated after plasma treatment. Figure 6 depicts possible chemical reactions between carboxyl groups and epoxy (Figure 6a), amines and epoxy (Figure 6b), and carboxyl groups and amines (Figure 6c). With a nonactivated surface, only epoxy and amine react with each other. However, the plasma treatment generates oxygen-based polar functionalities such as ether (–O–), hydroxyl groups (–OH), carboxylic acids (–COOH), and esters (–COOR) [22]. Unreacted epoxides and functional groups react further to form strong chemical bonds with the coating layer at the CFRP interface.

#### 3.2.1. Surface Morphology

**Figure 7:**
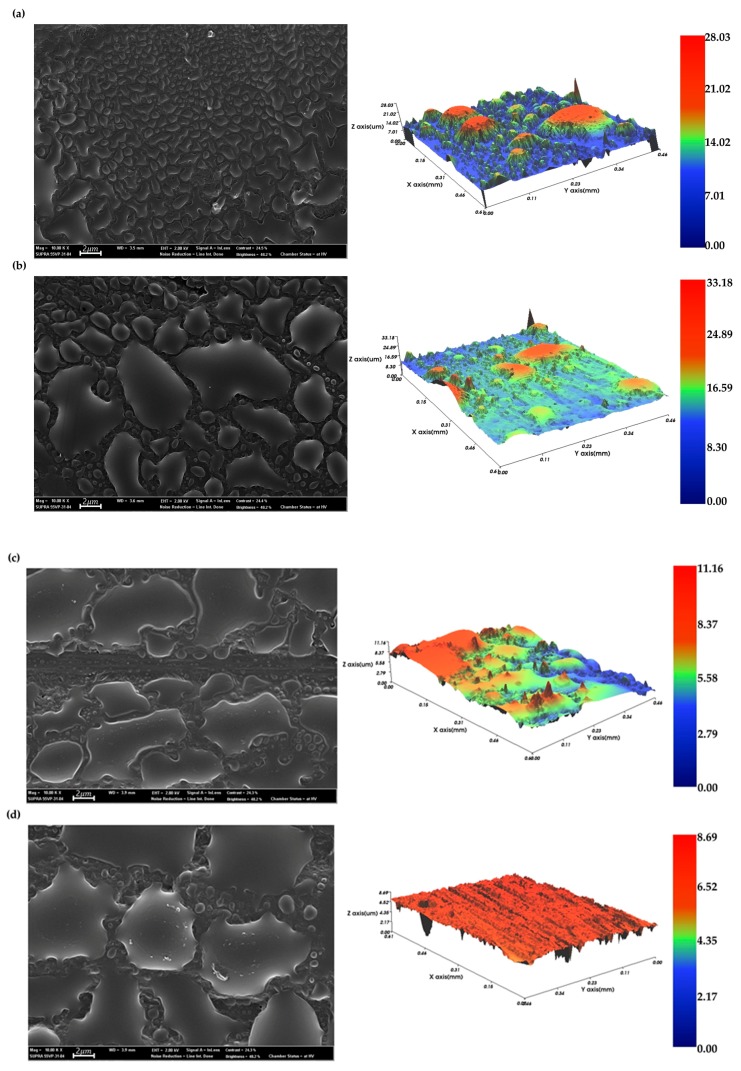
Scanning electron microscopy (SEM) and noncontact 3D microsurface profiler image of epoxy modified primer (EMP) coating: (**a**) P0 + EMP, (**b**) P1 + EMP, (**c**) P5 + EMP, and (**d**) P10 + EMP.

The surface morphology of the EMP coating was examined by scanning electron microscopy (SEM) and noncontact 3D microsurface profiling. Figure 4 shows that without any surface treatment, CFRP showed hydrophobic characteristics. Figure 7 shows that the wettability of the EMP coating changed with increasing number of plasma treatments, while Figure 7a shows that without any plasma treatment, the pristine CFRP is hydrophobic and P0 + EMP did not form a uniform coating layer. The SEM image shows that the EMP coating did not spread and formed numerous shapes on the surface. The noncontact 3D surface profiling also showed that the coating was partially confined in the case of P0 + EMP. The plasma treatment became more hydrophilic and the wettability improved as shown in Figure 4 and Figure 7. The observation of EMP spread gradually improved through SEM images and surface profiling images. The nonuniform surface improved gradually. In the case of the last P10 + EMP, the noncontact image shows the formation of a uniform coating layer.

#### 3.2.2. EMP Coating Adhesion Properties

SAICAS equipment was used to confirm the binding forces between the CFRP and EMP coating layers. First, a diamond blade was used to vertically cut the coating layer at an angle of 60°. The diamond blade cut down vertically and then, after reaching the CFRP interface, cut horizontally. The experiment was conducted to check the interface peel force between the CFRP surface and EMP coating. This vertical cut was mainly used for depth profiling to measure the internal forces of the coating layer. The vertical load also indicated the thickness of the coating layer. As the vertical load approached 0 N, the diamond blade approached the CFRP surface. The EMP coating depth showed that P1 + EMP of 11 μm, P5 + EMP of 10.9 μm, P0 + EMP of 9.6 μm, and P10 + EMP of 8.1 μm were measured. P10 + EMP showed the best wettability and the thinnest coating layer [23].

**Figure 8:**
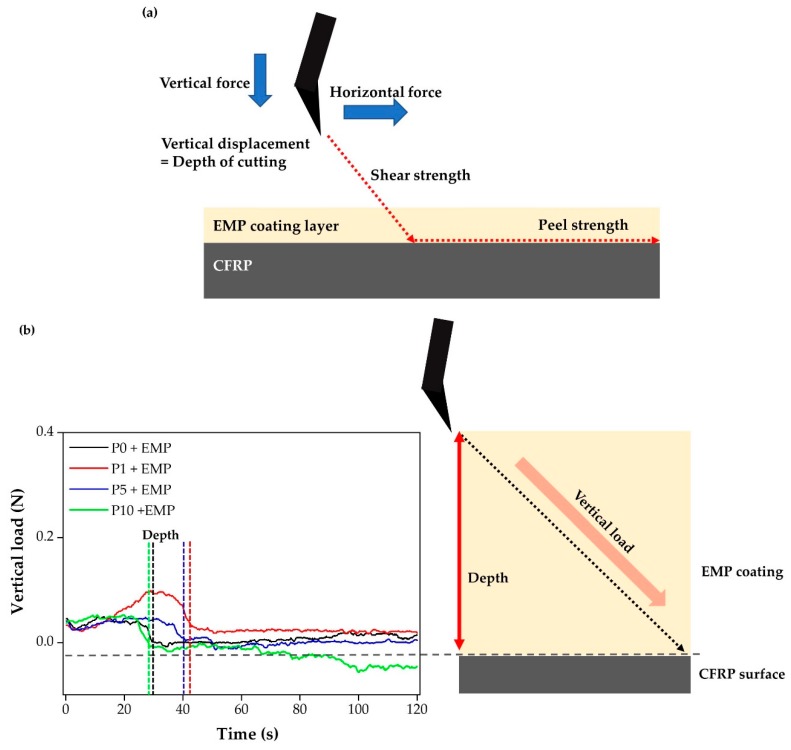

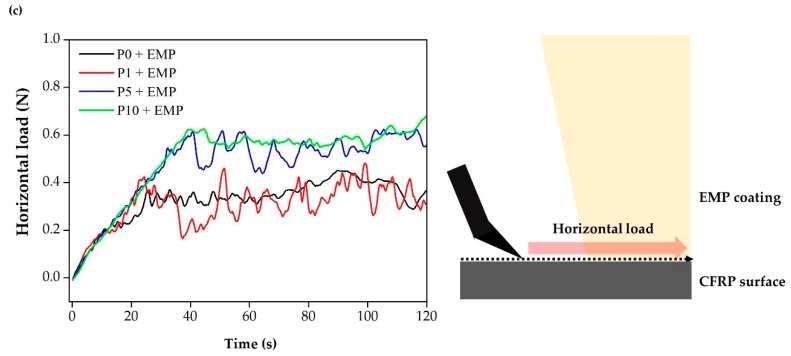
(**a**) Schematic of surface and interfacial cutting analysis system (SAICAS) operation, (**b**) vertical load of epoxy modified primer (EMP) coating, and (**c**) horizontal load of EMP coating.

The horizontal force was measured during the peeling of the EMP coating from the CFRP surface. Figure 8 shows that the horizontal forces at P0 + EMP and P1 + EMP were 0.4 N. As the number of plasma treatments increased, additional chemical bonds formed, as indicated by P5 + EMP and P10 + EMP, which showed a horizontal force of 0.6 N. With zero or a single plasma treatment, the thickness of the coating layer was not constant, and the graph of horizontal force fluctuated significantly. As the number of plasma treatments increased, SAICAS showed that stronger chemical bonds and more consistent coating layers formed.

### 3.3. Mechanical Test and Fracture Analysis

**Figure 9:**
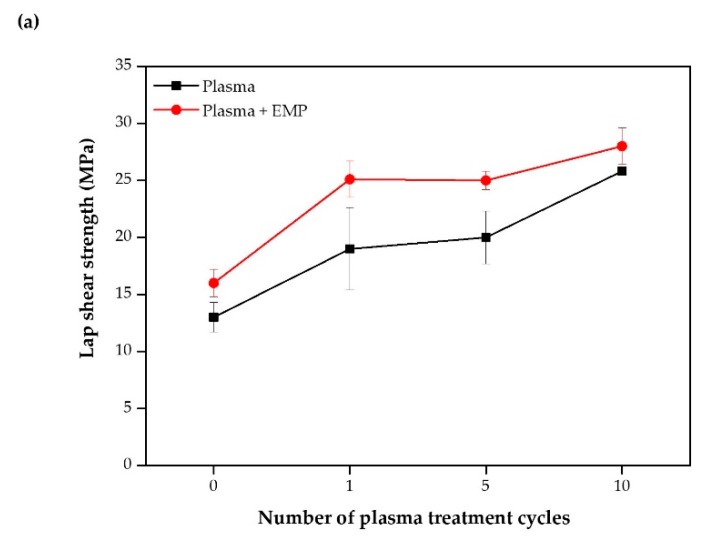

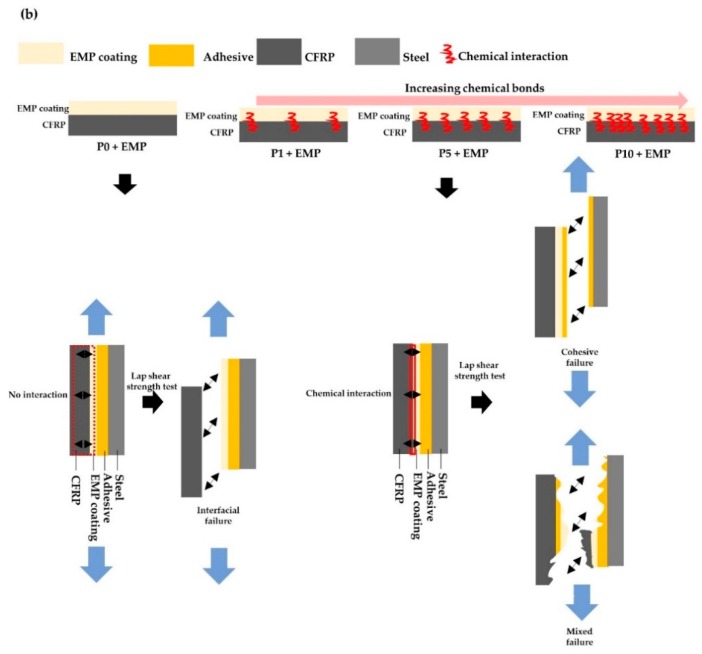
(**a**) Effect of the number of plasma treatments and epoxy modified primer (EMP) coatings on the lap shear strength and (**b**) schematic of interactions during lap shear strength evaluation.

The lap shear test was used to check the EMP coating effect via measuring the mechanical strength. Given the use of CFRPs with dissimilar materials in automobiles, the adhesion of CFRP to steel is important. Figure 9a shows the effect of plasma treatment changes on adhesive bonding. In addition, increased mechanical properties were observed with the plasma + EMP coating. With P0 + EMP, Figure 9b shows no significant difference because no chemical bonding occurred; the epoxy was a simple coating layer with no chemical bonding to the CFRP surface. 

The lap shear results showed no significant differences from the pristine state. Figure 9b shows that as the number of plasma treatments increased, the number of reactive species also increased, thus forming more chemical bonds. The bonding between CFRP and the coating layer strengthened, increasing the lap shear strength; this is unlike P0 + EMP, which had no chemical bonds. The interface of the coating layer was weaker than that of the adhesive. When the interface of the coating layer is stronger than the adhesive, the CFRP is destroyed or the middle of the adhesive layer is destroyed. The result of the lap shear strength measurement is important, but observing the fracture shape is even more significant after measurement. The adhesion performance with the interface can be determined according to the fracture shape. In P0, the adhesive strength was 13 ± 1.3 MPa, but that of P10 + EMP increased to 28 ± 1.6 MPa.

**Figure 10:**
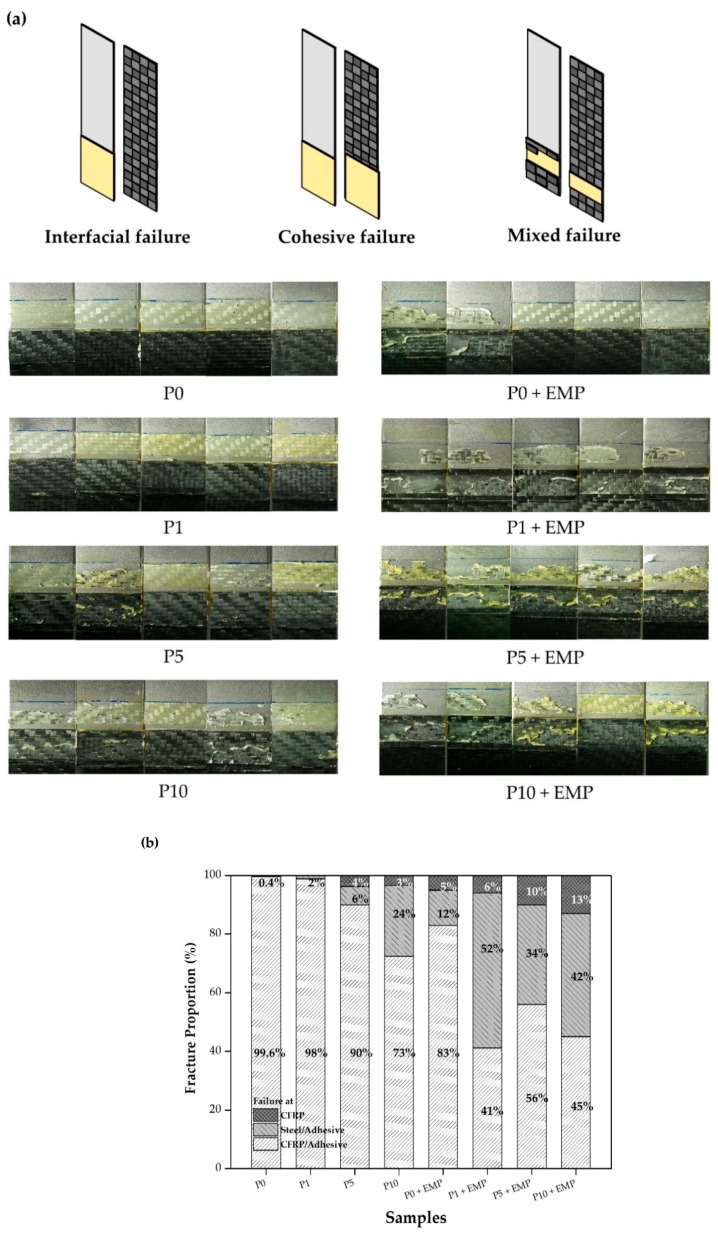
Observation after lap shear strength measurement: (**a**) Fracture shape and (**b**) fracture proportion.

The adhesive fracture surfaces were analyzed after lap shear testing using ImageJ image processing software. A lot of information can be obtained from the adhesive fracture shape. The failure shape and lap shear test were correlated and analyzed. In the case of P1 + EMP and P5 + EMP, the lap shear data were similar: 25.1 and 25 MPa, respectively. When we observed the shape of the fracture, a big difference was seen. The CFRP substrate fracture rate changed from 6% to 10%. The higher substrate fracture rate indicated that the bonding force between the substrate and the adhesive was stronger. The EMP coating and simple plasma treatment also showed significant differences in failure mode, as depicted in Figure 10a. For P0 to P10, sample failure generally occurred at the interface, except for a few samples. This means that simple plasma treatment shows imperfect adhesion performance when bonding steel to CFRP. Most of the samples of P1 + EMP to P10 + EMP showed mixed-mode failure and cohesive failure, except for P0 + EMP, which showed the need for surface treatment [24]. 

Figure 10b shows the fracture proportion. In the case of simple plasma treatment (P0, P1, P5, and P10), the rate of adhesive falling off from the CFRP interface was high. This indicated that the adhesion between the CFRP interface and the adhesive force was not strong enough. Except for P0 + EMP, the P1 + EMP, P5 + EMP, and P10 + EMP samples showed significantly lower interfacial failure rates in CFRP. The CFRP substrate breaking rate also increased by 6%, 10%, and 13%, respectively. An increase in the substrate breaking rate indicated that the interfacial adhesion between CFRP and the adhesive was extremely strong, i.e., the interface and adhesive bonding was very good, leading to the breakage of the CFRP.

## 4. Conclusions

Experiments were performed to improve the adhesion between dissimilar materials (steel and CFRP) in order to reduce the weight of automobiles. CFRP can show poor adhesion in a pristine state; however, the adhesive performance of different materials can be improved through surface treatment.

Plasma treatment was performed to generate activators (oxygen-containing functional groups) and improve the wettability of the surface; increases in oxygen-containing functional groups (C–O, O–C=O) were successfully observed (P10 > P5 = P1 > P0) to increase the carboxyl group. This increased carboxyl group cross-linked with epoxy.

SAICAS was used to check the degree of chemical bonding between CFRP and the coating layer. Lap shear test results also showed that adhesive strength increased with increasing numbers of plasma treatment cycles. P10 + EMP showed the strongest chemical bonding and highest horizontal force. 

In the lap shear test, P10 + EMP surface treatment showed an increase of 115% compared to pristine CFRP. In terms of the fracture shape, the CFRP interfacial failure changed to adhesive failure and mixed-mode failure. This plasma + EMP coating process can solve the issue of the endurance of the plasma treatment. In addition, automotive parts fabricated by plasma + EMP coating process will be used frequently because it can be applied directly without surface treatment in the automotive body process.

## Figures and Tables

**Table 1:** Epoxy modified primer composition.

Epoxy Modified Primer			
Material	Composition	Equivalent Weight (g/eq)	Form
YD-128 (Kukdo Chem.)	Bisphenol-A epoxy	187	Liquid
Dyhard 100 s (Alzchem.)	Dicyandiamide	21	Powder
Dyhard UR500 (Alzchem.)	Substituted urea	3	Powder
Ethyl Acetate	Solvent		Liquid

**Table 2:** Adhesive composition.

Adhesive			
Materials	Composition	Equivalent Weight (g/eq)	Form
KSR-177 (Kukdo Chem.)	Bisphenol-A Type	205	Liquid
G-5022 (Kukdo Chem.)	Polyamide	175	Liquid
